# Special Issue: Place, Space, and Mental Health

**DOI:** 10.3390/ijerph20065055

**Published:** 2023-03-13

**Authors:** Tytti P. Pasanen, Anna Kajosaari

**Affiliations:** 1 Faculty of Social Sciences, Tampere University, 33014 Tampere, Finland; 2 Environmental Health Unit, Finnish Institute for Health and Welfare, 70701 Kuopio, Finland; 3 Department of Built Environment, School of Engineering, Aalto University, 02150 Espoo, Finland; 4 Institute for Urban and Regional Research, Austrian Academy of Sciences, 1010 Vienna, Austria

Global megatrends, including climate change and urbanisation, are shaping and changing how we live in the future. Multiple aspects of our living conditions—environmental and societal—are subject to change regardless of where we live. Recently, the COVID-19 pandemic rapidly placed us in an unexpected new situation that dramatically affected our daily lives. These changes have had and will continue to have an effect on our mental health and well-being [1,2]. With the global increase in mental health issues, deeper understanding of how place and space affect our mental health is therefore urgently needed.

This Special Issue, “Place, Space, and Mental Health”, called for contributions dealing with the various ways in which human mental health is affected by its physical surroundings. We were particularly interested in innovative research methodologies addressed at tackling topical issues regarding the role of place and space for mental health. The seven included papers all provide new knowledge on how mental health is affected by place and space at different scales, and, more broadly, how these processes can be considered in the urban planning of specific types of places and landscapes.

The included collection of articles is diverse in terms of topics, methodologies, and environmental domains. Three papers are related to environmental crises and the ways the environment can help us cope when faced with unusual situations. Regarding the recent COVID-19 pandemic, Bernat et al. [3] assessed students in Lubin, Poland and found that recreational areas, especially those with nature, scenic views, and therapeutic values, gained importance among students during the pandemic. Scrima et al. [4] examined the perspective of employees in a COVID-19 vaccination centre and found that their appreciation of art at the workplace was associated with less work-related exhaustion. Chan et al. [5] examined dilemmas over relocation after a natural disaster, flash flooding, in Ellicott City, USA. Their study showed how leaving one’s home after a crisis is a complex dilemma involving communal, historical, environmental, emotional, and economical considerations.

Four papers focus on the connections between mental health and everyday places and spaces. Swahn et al. [6] presented views from an underrepresented population in academic research, i.e., young women residing in urban slums in Kampala, Uganda. The participants identified social and physical environmental features in their neighbourhoods that elicited both positive and negative emotions, and relatedly, reflected on their mental health. The papers by Zhang et al. [7] and Reece et al. [8] focused on immediate responses to outdoor scenes with the presence of greenery and historical or heritage value. Zhang et al. [7] found that the proportion of grass and water in a heritage landscape predicts emotional responses to historical landscapes and observed that deep-learning facial recognition was able to detect more subtle changes in valence than self-reported measures. Reece et al. [8] found that the presence of historical elements—but not greenery—was associated with self-reported emotional responses to outdoor urban settings, whereas brain activity responded more clearly to greenery. Focusing on transitional spaces between indoor and outdoor settings, which have received little research attention so far, Li et al. [9] concluded that viewing curved elements in these spaces reduced both physiological and psychological stress more than viewing linear elements.

Overall, this collection demonstrates the diversity of current research methods in the analysis of the interrelations between place, space, and mental health. Two articles are based on the quantitative assessment of cross-sectional surveys targeting specific groups: university students in Lubin, Poland [3] and staff of vaccination centres in Palermo, Italy [4]. Diverse qualitative methods were applied via in-depth interviews with adults who had encountered a natural disaster, i.e., a flash flood, in Ellicott City, USA [5] and via photo-elicited focus group discussions for young women living in slums of Kampala, Uganda [6]. Three experimental studies in this collection utilised state-of-the-art technological tools in either developing environmental stimuli (immersive virtual reality scenarios [9]), processing environmental stimuli (Google Street View images analysed with deep learning algorithms [7]), or measuring responses to environmental stimuli (brain activity by EEG [8]).

In addition, this collection exemplifies the different physical and social domains and scales that are present in our everyday lives. The importance of public open and recreational spaces for the promotion of mood and mental health was examined by Bernat et al. [3], Reece et al. [8], and Zhang et al. [7]. Swahn et al. [6] and Chan et al. [5] assessed the role of communities in the wider environmental and social context. Scrima et al. [4], instead, focused on workplaces, where people spend large amounts of their days, and Li et al. [9] on transitional spaces between indoor and outdoor environments where visitors pass several times a day but only for brief amounts of time. Yet, Li et al. [9] showed that these places and their design can affect short-term stress responses.

With this collection, we hope that readers will gain new perspectives into how our everyday mood and mental health rely on and are affected by our surroundings in terms of various spatial and temporal scales. Understanding these interactions remains a topic open to active inquiry and experimentation among diverse research fields addressing mental health in place.
